# American Dental Association

**Published:** 1891-11

**Authors:** 


### Reports of Society Meetings.

### AMERICAN DENTAL ASSOCIATION.

*August 4, 1891.—First Day.—Evening Session.*

Dr. Smith, the chairman of Section VI., "Physiology and Etiology," desires to present the following report:

"At the annual meeting two years ago, this Section was empowered to make an examination of prehistoric crania, and an appropriation of money was made for that purpose. At the last meeting Dr. J. R. Patrick was selected to act as curator of the proposed examinations. Partial examinations of collections of crania in Washington, Boston, and the curator's own valuable collection have been made. A brief report of the progress made in the work of tabulation will be given by Dr. Patrick. The members of Section VI. express the hope that the work already done will meet the approval of the members of this Association, when they shall have had an opportunity of examining the same.

"I should state here, perhaps, that the amount of money appropriated for this investigation, as you remember, was five hundred dollars, and fifty dollars of that sum had been used before Dr. Patrick began his investigation. Since then the whole sum has been expended, and I think there is a small deficit of about ten or eleven dollars over the sum appropriated. The Section, therefore, thinks that another appropriation for the continuance of this work would be proper.

"Dr. Patrick will present his report this evening, and then you will be better able to judge of the fitness of expending the money.

"We have also to report that the following papers have been submitted to the Section: 'Mouth-Breathing not the Cause of Contracted Jaws or High Vaults,' by Dr. Eugene S. Talbot, of Chicago; 'Physiological Action of Obtundents,' by Dr. L. E. Custer, of Dayton, Ohio; 'Diseases of Oral Mucous Membrane,' by Dr. Patterson, of Kansas; 'Dentition of the Felidæ,' by Dr. Alton H. Thompson, of Topeka (this will be read by Dr. Peirce); 'Phagocytosis,' by Dr. H. A. Smith, chairman of the Section.

"I would also state here that a paper was handed the Section by Dr. Waters, of Boston. The paper was passed into the hands of Dr. Barrett, and I have not had a report from him; but if it be read, I would ask that it be by title only,—at least for this evening.

"After the reading of the short report on Dental Implantation, by Dr. Ottofy, I think it might be in order to ask Dr. Talbot to read his paper.

"The Section still continues its observations regarding the once popular dental implantation. At the present time the Section is prepared to make the following statements regarding the implantation of teeth:

"*First.* In properly selected cases, transplantation is successful for from three to five years.

"*Second.* No other operation in dentistry equals in length, beauty, service, appearance, and perfect comfort that of successfully implanted teeth.

"*Third.* No disease has ever been transmitted by implantation.

"*Fourth.* A combination of transplantation and implantation will often enable a dentist to make good use of a tooth lost by some cause. We believe the practitioner is justified in performing this operation in suitable cases."

### MOUTH-BREATHING NOT THE CAUSE OF CONTRACTED JAWS OR HIGH VAULTS.

BY DR. EUGENE S. TALBOT, OF CHICAGO.

Mouth-breathing was unknown to the early races, and is not observed among the present pure races nor modern civilized races. This may also be said of the deformities of the maxilla and teeth.

In an essay upon the subject of "The Influence of Adenoid Hypertrophy at the Vault of the Pharynx upon the Development of the Hard Palate," read before the New York Odontological Society, November 16, 1890, by Dr. D. Bryson Delavan, the author speaks of mouth-breathing as a cause of many deformities.

Other authors mention the fact, too, that sleeping with the mouth open produces tension of the buccinator muscle, causing the jaws to contract, and have suggested different theories by which that pressure brings about this peculiar form of deformity.

I will direct attention to a few facts as they have been presented to me in the constant study of deformity of the jaw and teeth for the past fourteen years, and you, gentlemen, shall be the judges whether mouth-breathing has anything to do with contracted arches or not.

In the first place, let us consider the part involved. The superior maxillary bones are fused at the median line. The outer surfaces have upon their borders an alveolar process. The maxillary bones proper are made up of tense, compact tissue, and are so arranged as to best resist force. They are also for the attachment of muscles and the resistance of force in masticating food.

The hard palate does not assume the normal shape until the twelfth year, or after the teeth are all in position. The vault may be high or low, ranging from one inch above the margin of the alveolar process between the second bicuspid and first permanent molar down to one-quarter of an inch from the same point.

The alveolar process is made up of soft, cancellated structure, and is solely for the purpose of protecting the germs of the teeth before they have erupted.

From the time the teeth make their first appearance until they are finally shed, the alveolar process has developed and been absorbed three distinct times.

The buccinator muscle is composed of striated muscular fibres, and is therefore under the control of the will. Its function is for the purpose of compressing air in the act of blowing, whence its name is derived, and also for the purpose of carrying and holding the food under the teeth during mastication.

There are many cases of contracted arches where mouth-breathing does not exist. Mouth-breathing frequently commences very early in life. Contracted jaws, on the other hand, never commence until the seventh or eighth, and sometimes the tenth year. Contracted arches are of two kinds,—namely (Fig. 1), V-shaped and (Fig. 2) saddle-shaped. All the other varieties are modifications and blendings of these two kinds.

In the V-shaped arch, commencing at the first permanent molar, there is a gradual narrowing of the teeth and alveolar process towards the median line. There is also a protrusion of the teeth and alveolar process, and not the jaw.

In the saddle-shaped arch, the bicuspids are carried inward, and the deformity is invariably situated between the first permanent molar and the cuspids. The contracted hard palate is always associated with the V-shaped variety.

In mouth-breathing the lower jaw usually drops only sufficiently for the passage of the same volume of air as would pass through the nasal cavity, which is only about half an inch. Old people often sleep with the mouth open, and frequently to the fullest extent; but this deformity of the jaw and teeth never occurs after the eruption of the teeth, say the twelfth or fifteenth year.

As to the buccinator muscle, it extends anteriorly to the first bicuspid only, and therefore it can produce no effect upon the V-shaped variety of deformity, in which is also found the contracted vault.

For years it has been demonstrated by dentists in regulating teeth, that it is very rare for the apices of the roots of teeth to move when pressure is put to bear upon the crowns of teeth for the purpose of regulating them. This being the case, teeth having no roots are less liable to move than teeth with short roots like the lateral incisors and bicuspids. Since in the moving of a tooth the greatest change which takes place is at the neck, it stands to reason that the greatest absorption and deposition of bone takes place at that point.

I have shown that the first permanent molar and the teeth posterior to that are never involved. I have also shown that the centre of the muscle in both directions is located at this tooth. How is it possible, since all the teeth are covered by the muscle upon one side, that half are carried inward and the other half remain normal?

Again, if mouth-breathing is the cause of contraction, both sides must contract alike, which never occurs.

The pressure of the tissue upon the crown of teeth is not sufficient to affect the alveolar process through the roots of the teeth, but even if it could modify those spongy structures, its force would stop there, and would not extend to the osseous vault, bending it out of shape.

The changes which take place in bone are not the bending in at one place and forcing out at another, but are an absorption and deposition of bone at the point of pressure.

It would be as impossible to produce pressure sufficient to break the dental arch as it would be for the weight of a building to break the arch of a door or window.

The V-shaped arch can never occur upon the lower jaw if the teeth articulate normally, because these teeth strike inside of the upper, and are thus prevented from moving forward. If we take three thousand models of the upper jaw, and arrange them in groups according to the forms here represented, and then examine very closely the arrangement of the teeth in each group, we will be unable to find any two alike in any group,—thus showing that any external force acting upon the root from the outside could not possibly be the cause of contracted vaults.

The maxillary bone never protrudes in front in this class of cases. It is only the alveolar process which is carried forward by the projecting teeth. The only tissues involved in those deformities are the teeth, on the one hand, and the alveolar process on the other.

It would be useless for any one to say that mouth-breathing is the cause of one case of V-shaped arch in every twenty, and that some other cause produces the rest of the deformities. We must have a law which will work in all varieties of contracted arches.

The following table will explain the difference in the height of vaults both in normal and defective jaws:

| | |
|---|---|
| Normal jaw, average | .58 of an inch. |
| Saddle-shaped arch, average | .60 " " " |
| V-shaped arch, average | .55 " " " |
| Semi-V and semi-saddle-shaped arch, average | .56 " " " |

It seems, then, that what has been considered a change in the height of the vault in narrowed and contracted jaw is only an imaginary one, the contraction conveying the impression that the vault is much higher than it really is.

In order to strengthen further the views herein suggested, I have taken impressions of the mouths of a large number of mouth-breathers, and have secured models of the same.

Dr. Talbot then gave about forty illustrations of different cases.

DISCUSSION.

*Dr. Watkins*, New Jersey.—I would like to ask the gentleman if all these models are taken from the mouths of mouth-breathers?

*Dr. Talbot.*—Yes.

*Dr. George J. Fredericks*, New Orleans.—I was not here when the paper was read, but I heard the close of it. I think these measurements are to prove the difference between mouth-breathers and nose-breathers. Is not that the point?—that mouth-breathing has a tendency of changing the jaw?

*Dr. Talbot.*—I did not try to make any point. I made these measurements to see if there was a difference between contracted arches and high vaults of mouth-breathers, and I have shown by the examinations that the vaults are lower on an average in contracted arches than in normal arches.

*Dr. Fredericks.*—Has the normal arch been established?

*Dr. Talbot.*—I should think that six thousand four hundred cases ought to establish it.

*Dr. Fredericks.*—That is exactly the condition of health and disease; because, when you want to find what health is, then you are on a doubtful subject. It is something that you do not know. Where one man is considered healthy, in comparison with another he may be a sick man. It is the same in regard to the measurement of the roof of the mouth. If you take even six thousand four hundred cases, you cannot establish a normal condition, in my estimation.

*Dr. Abbott.*—I want to congratulate Dr. Talbot upon what seems to me a very successful outcome of his work in this direction. It seems very evident that he has established the one fact, that mouth-breathing does not produce the contracted arch. This is to me rather an interesting and a valuable piece of information. Although I am not in that line of study, I see more or less of it; and of course am very much gratified indeed at this paper, and the results of the work that Dr. Talbot has done.

*Dr. Fillebrown.*—I have one suggestion to make in corroboration of Dr. Talbot's paper. I think if any person present will drop his jaw down so that it is as wide open as any mouth-breather would have it, he would find that there is abundant room for the air to pass between the teeth. You will also find that the lower jaw lies far away from the teeth, and does not affect them in any way. When the cheeks are sunken in, from old age or from loss of the teeth, it is not because the teeth themselves are gone, but it is because the jaws drop together, thereby making the cheeks depressed. Consequently, I say that that circumstance is abundant proof that the pressure of the muscles on the maxilla does not tend to contract the arch in its lateral diameter.

*Dr. Horton*, Cleveland.—I have read many articles on the subject of mouth-breathing being the cause of contracted arches, but I think we can have nothing more conclusive than the refutation that has been made by the paper just read. I am happy to say that I believe the doctor has succeeded in perfectly overcoming the position that has been taken heretofore.

*Dr. Watkins.*—I understood from the doctor's paper that he stated that the contraction of the arch was not caused after the teeth had erupted. I would like to ask him if he has found that mouth-breathing was the cause of any contraction of the arch before the permanent teeth were erupted, or during their eruption.

*Dr. Talbot.*—Being a graduate of medicine, I have had an opportunity to examine into the early causes of thumb-sucking and mouth-breathing, and I have found that babies sometimes breathe through their mouths within two or three weeks after their birth. Yet I do not think we see a case of contracted arch with the first set of teeth. We do not see contracted arches until the eighth year, and the contraction ceases after the teeth are erupted. It takes place from the time the teeth erupt, when they come through.

Motion here made that members of the profession from other countries be invited to take part in the discussions. Carried.

*Dr. Peirce.*—Mr. President, I must dissent somewhat from the conclusions of the essayist. I think he has misstated the case when he says that mouth-breathing has been considered the main cause for contracted arches. It has been considered one of the factors, and the statement he has made here this evening certainly is an argument in favor of that. The fact that deciduous teeth are never contracted by mouth-breathing is simply due to the fact that the mouth is shaped in early life, and the teeth that are being erupted are short, and there is little mechanical force brought to bear upon them, so that they cannot be forced into the jaw during their short period of development. He stated also that the mouth changed, or became contracted, from eight to twelve years, and that is the period that these bicuspid teeth, which are among the longest teeth we have, are gradually coming down, and the pressure brought upon those teeth by the cheeks does have a tendency, while they are being developed, to force the crowns a little into the mouth, and hence produces a narrowed arch.

The fact that the change never occurs after twelve years shows that the development has taken place while the teeth were being erupted, so I simply claim that this mouth-breathing, or the fact that the mouth is kept open while the child is at rest, is one of the factors in forcing the sides of the mouth together. He says if this were the cause, that both sides would be influenced alike. Not necessarily, because the pressure may be greater on one side than the other. On one side the child is lying, and on the other is the weight of the cheek. There is always a difference in weight. Because the result is not the same on both sides, it seems to me, is not an evidence that there is not some influence of that kind at work. The evidence that has been produced in favor of it is what he has stated to-night,—that from the ages of eight to twelve years is just the period that the change takes place, and only then; and that is the period when these bicuspid teeth are being slowly developed.

*Dr. Horton.*—These departures from the normal shape of the mouth are confined mostly to the cultivated races. I would state that they are very much more rare among negroes than among whites in this country. It is a comparatively rare occurrence to find a negro with a high or deformed arch, or with irregularities of the teeth even; so that I take it that the doctor's observations are correct, and I wanted to emphasize that single idea in his most excellent paper. I may not have understood, as my hearing is a little dull, the reading of the paper thoroughly. If I did, he undertook to say that the alveolar processes were developed, and that their office was to some extent to protect and cover the germs of the teeth. I think alveolar processes never make their appearance until the crowns are free from the bony structure in their development, and that the alveolar processes are developed with the growth of the root of the tooth. The alveolar processes are not developed, except as the root of the tooth is developed.

*Dr. Truman.*—I would like to ask Professor Peirce this question, If the bicuspids from eight to twelve years of age are liable to be drawn into the mouth by the action of the buccinator muscle, why is it that the deciduous molars are not drawn in? And if a deciduous molar is drawn in, why of course it will drag in the permanent bicuspid as it is developed between the roots of the deciduous molar teeth. Several years ago I took the same position that Dr. Fillebrown does, that it was impossible for those muscles to act upon the teeth and contract the arch, and I am gratified to-night to find that this very absurd theory of mouth-breathing producing these irregularities has been settled for all time.

*Dr. Peirce.*—I simply would state that, looking at it mechanically, the deciduous molars are very short, and they are very quickly erupted, compared to the length of time that the bicuspids are occupying their permanent places, and therefore there is not nearly the mechanical influence brought to bear upon deciduous molars that there is upon the permanent bicuspids. Now, if the argument brought out by the doctor in his paper this evening be correct, then we would suppose that softening of the muscles of the cheek would have no influence; yet I am certain many gentlemen have seen a slight aneurism on the cheeks that has forced the bicuspid and the molar on to the palatine surface. I have had two cases of that, and one a very marked one, where the aneurism of the cheek was as soft as it could be, and yet its presence did displace those teeth.

*Dr. Abbott.*—In reference to the development of alveolar processes, the formation begins at the time of the development of the crown, and when the crown of the permanent tooth is fully formed, the alveolar process is in considerable growth. This I have observed in many cases.

*Dr. Patrick.*—Mr. President, I have very little to say in regard to Dr. Talbot's paper, only so far as measurements are concerned and averages. Now, I have always admired energy,—even the energy of a squirrel in a cage; but the utility of that energy spent in that direction I never could admire. Averages are not scientific facts. They are speculations. You take ten thousand measurements of any portion of the human body. You divide it equally between each one. You have then an average. The very moment you make one more such measurement, you destroy the whole average! Take one thousand human superior maxillas and measure them; find the average. You take one more, and that either measures a little more or a little less, and the average is useless for any purpose whatever. Then I object to the manner in which these measurements have been made, irrespective of race, irrespective of what people they belong to. You do not even get an average of one single race of people. One more measurement, and the whole fabric is upset. One instance of a large jaw or a small jaw destroys the whole thing.

Therefore an average is not a scientific fact. It answers a purpose in some instances for speculation; for instance, the life insurance agent can deal with averages,—the average age of man; but he leaves an immense margin in taking a risk. It is not scientific; it is speculative. It is gambling on the life of man.

That is not all. While an average is not a scientific fact, it is worse, still worse. It destroys individuality. It is destructive of the study of individuality. It puts aside and renders classification impossible, if you take an average for a guide. Men have tried that for the past one hundred and fifty years in scientific matters, and have always abandoned it. I shall not occupy your time by reciting prominent instances of it.

Dr. Morton, one of the most energetic, well qualified, thoroughly educated Philadelphia gentlemen, says it is a speculation. Dr. Morton made a splendid collection of primates. He spent his whole life and energy in establishing it. He deserves to be called the father of primeology, if it ever becomes a science.

So much for averages. So much for measurements. Measurements are very useful. I will not gainsay anything that mensuration will do. Engineers would be lost without it, but where it tends to averages, it amounts to nothing. Where it tends to mark the bounds of individuality, it is valuable, because it gives you the true key to classification.

A celebrated writer has discovered a system of measurement of the human body for the purpose of identification of criminals. The system of measurement rests on the fact that there are no human beings on the face of the earth who are exactly alike. That is why it is valuable; so that if he measures one individual, he can always be identified from the single fact of the dissimilarity of all others. It is valuable in no other sense. Its great value rests on that fact. There are no two human beings, even of one family, who are exactly alike. In taking the measurements of enlisted men, it is a good thing to use that method to recover deserters; but still they may disfigure themselves afterwards.

*Dr. Talbot.*—I will not attempt to answer Dr. Patrick at this time. I will take him when he reads his own paper. I want to speak of the remark made by Dr. Peirce, that the greatest contraction is at the second bicuspid tooth. If the buccinator muscle is the cause of that, the first permanent molar must be involved, because it involves the wisdom tooth; but we rarely find the first permanent molar involved in a contracted arch. Now, it would be impossible, in Fig. 4, for the muscle to produce that style of irregularity, because it could not bend sufficiently to produce that form. Again, if mouth-breathing were the cause, the first teeth would necessarily be involved, because the very point he makes shows that, the teeth being smaller when the alveolar process is in its constructive stage, the temporary teeth would be involved; but we never see it, and therefore it would be impossible for the buccinator muscle to produce contracted arches.

*Dr. Peirce.*—I did not say a word about the buccinator muscle. I said the tissue lying over the teeth.

*Dr. Talbot.*—The tissues could not be involved at all without there being a muscle that produced it. The mucous membrane could not contract.

Dr. Patrick then read the following report:

Mr. President, I have the honor to report the following: The work of examining the crania was intrusted to Dr. Andrews, of Cambridge, Massachusetts. He found it expedient to employ Mr. Newton, a physiologist, to assist him. Mr. Newton is a young man, a student. He found it necessary to devote his whole time for one month to the examination. The work has been carefully and faithfully performed by Mr. Newton, and vouched for by Drs.

Andrews and Knight. The following crania of different races were examined:

| | |
|---|---|
| Peru | 504 |
| Terra del Fuego | 1 |
| Mexico | 10 |
| Stony Grove Indians | 154 |
| California Indians | 191 |
| Nicaragua | 816 |
| Guatemala | 1 |
| Mexican | 2 |

The work of examining and tabulating the crania was intrusted to Dr. Peirce, of Philadelphia, with the assistance of Dr. Ives. They have examined one thousand and six human crania, which completes the work on the collection of crania in the Academy of Natural Sciences, Philadelphia. The work of examining the Patrick collection now in the rooms of the Historical Society at St. Louis was performed by Drs. McKellops, Fuller, and Wick, of St. Louis. The crania were taken from a burial mound situate in St. Clair, Madison County, Illinois. They number sixty-nine. A small collection of forty human crania has been examined and tabulated by Drs. Ottofy and Davis, of Chicago. The record of the finished work now in my possession was returned too late to make a report in regard to the condition of the teeth and jaws of the several collections. The collection in the Medical Museum has a record of one hundred, making a total of two thousand and ninety-six.

I wish to state here that all of these records were returned to me within a few days of my leaving for this place. Of course it was impossible for me to make anything like a report of the physiological or pathological condition of the jaws and teeth as recorded, but I think that I would be able, if you will allow me to do it, to make a record of what I have now on hand, in time for publication in the transactions of this society. I propose to give the record in such a manner that it would be read. I would take each race of men and give the condition of each skull in each race, so you could read it straight along without referring to tables. This is one of the records. (Exhibiting same, and passing it around for examination.)

There are one hundred in each volume. I have made provision for the tabulation of ten thousand. I do not think it would be more than half enough. I have quite a number of gentlemen from whom I have not heard. I expect to receive letters from Washington City shortly, calling my attention to other collections,—private ones.

Outside of the United States I have had several letters from people who are willing to work for the American Association in this investigation. Among them are Dr. Mitchell, of London, Dr. Ernst Shoeber, of Stockholm, and Dr. Van Marter, of Rome, Italy. There are a number of others, but I have not made any arrangements with them yet. I sent to the Peabody Museum, which was founded by Peabody at the instigation of his nephew. I think they will have, before we get through with this investigation, the largest collection in the world, and the classification is better than any I have met, because it is classified according to the geography of the country they were taken from.

It would be necessary, in order to prosecute this study properly, to compensate some of the gentlemen who do this work. I think it is wrong to expect them to do it for nothing. One or two men who are deeply interested made the suggestion that they would use their own assistants, knowing that they could not spare the time themselves. A man must take some one with him to assist him in carrying the skulls to and from the cases to the tables, and those assistants must be paid. Probably that would be Dr. Smith's part of the report. I want to say that before we meet again I will have a circular out in every State that has a State society. The Missouri State Association has already contributed fifty dollars; Georgia, twenty-five dollars, and so on; so far as I have heard from them, and so far as I have asked, none have refused.

Moved that the report be accepted as a report of progress. Carried.

### PHYSIOLOGICAL ACTION OF OBTUNDENTS.

BY L. E. CUSTER, DAYTON, OHIO.

This paper has been written with a view of making clear and harmonizing a few conflicting ideas regarding the action of obtundents of sensitiveness of the dentinal fibril.

Owing to conflicting theories, it will be fair to assume that this tissue, whose sensibility we desire to obtund is of the nature of simple protoplasm, with (in this instance) an exalted sensitive function. It contains albumen, and this is therefore coagulable; it is made up largely of water and may be desiccated; it is encased in a tube, and when it does not entirely fill this tube, a fluid fills this space. This fluid may be removed, and the temperature of the fibril may be reduced. These are the conditions upon which we are to work.

The dentinal fibril does not possess a blood circulation, and on that account the nutritive movements are slow; so slow, that any agent, which produces local anæsthesia in other parts in a few minutes, will be very slow in acting to any depth in the dentinal fibril, —so slow, indeed, that we need hardly look in this direction for a practical agent. Cocaine, as a typical representative of this class of agents, has not been effective for this reason.

Any agent which is to act upon the sensitive function of the dentinal fibril must be more powerful than cocaine; it must not coagulate albumen; it must have an affinity for water; it must possess a penetrating property and insinuate itself into the tubule, for it will not be carried in by circulation, and but very slowly by any nutritive movement. Until we find an agent possessing these qualities, we need not expect to obtund sensitive dentine by suspending the fibril's irritability.

The most satisfactory results have been obtained in other directions. The dentinal fibril has a definite composition. All the proteids are in a definite proportion, and we have reason to believe that the proportion is very delicately balanced. If the structure of the fibril be changed, its irritability ceases, and it can no longer communicate.

The change of structure most easily accomplished is the coagulation of its albumen and the withdrawal of its water. If the albumen is coagulated, it is as effectual in checking neural movement as though the albuminous ingredient had been withdrawn; indeed, the coagulation of albumen itself renders it harder, and its presence would prevent any exhibition of life more readily than if it were removed.

The coagulation of albumen, unless produced by heat, is somewhat self-limiting, so that, since heat of 160° F. is not allowable for sensitive dentine, the action of present known coagulants is not entirely satisfactory. Before coagulation can be a practical success, we must have an agent which penetrates to some depth.

Penetrating escharotics, such as arsenic and the like, since they burn the tissue beyond recovery and endanger the pulp, should not be considered. The other method of changing the structure of the dentinal fibril is by the removal of the water.

The water is in a twofold relation with the fibril, that which is a constituent of the fibril itself, and that which fills any inequalities between the fibril and Neuman's Sheath. One is a constituent, and the other a condition.

The water, which is a constituent, is always present; it is always found where there is life and motion; it is always a necessary condition for neural activity; it makes up three-quarters of the entire body, and about ninety per cent. of the dentinal fibril. Constituting such a large proportion of the fibril, it is evident that there would be a proportionate change in the structure and physical character if the water were removed.

The size would be decreased; the albumen, if coagulated, would become harder, and in this desiccated condition it would be practically dead, incapable of performing nutrition and function.

The water which fills any inequalities between the fibril and its investments may be accessory in the transmission of sensation. In this relation, it may transmit vibrations to the fibril or even to the fibriloblast.

One of the most delicate nerves of special sense—the auditory—receives its impressions through the endolymph in which its terminals float, so, if the water surrounding the fibril should be removed, the fibril's transmitting power may be affected.

The watery contents of the tubule are withdrawn by evaporation, and by bringing an agent in contact with it for which it has an affinity. Evaporation is produced by raising the temperature and subjecting the fibril to a current of air. This is practically accomplished by the repeated blasts from a hot-air syringe.

The water surrounding the fibril is comparatively easily abstracted, but to loosen the molecular grip in the fibril itself requires no force.

There are many agents which have an affinity for water, but not all are suitable for use in the cavity of a tooth.

As compared with coagulation as a means of changing the structure, dehydration is more thoroughly accomplished with the present known methods, and hence the results are more satisfactory; so much so, that I suppose the majority of operators use dehydration almost entirely as a means of obtunding sensitive dentine.

We have an agent which, when used on sensitive dentine, both withdraws the water and coagulates the albumen, and a more effective single medicament for sensitive dentine we have not. I refer to chloride of zinc,—not a fluid when its affinity for water has been satisfied, but a crystal which will deliquesce when placed in the cavity.

The last method by which the fibril-transmitting power may be lessened is by changing the temperature. Of all the obtundents of sensitive dentine, extreme cold is the most effective; not that it is any better than perfect coagulation or dehydration, but because the temperature of dentine may be reduced more thoroughly than the albumen may be coagulated or the water withdrawn in ordinary practice.

The reduction of the temperature is best accomplished by the use of volatile agents, such as sulphuric ether, chloride of methyl or nitrous oxide.

It will be observed that the fibril's function may be completely suspended by coagulating all the albumen, by withdrawing all its water, or by lowering its temperature to a certain point. Entire coagulation of its albumen is practically impossible at present, the entire removal of its moisture almost impossible, and the reduction of temperature is the only one which acts to any desired depth.

Recently there have been introduced three new methods, having the same principle of action, which are misleading,—viz., the thermal obtunder of Small, the Milton obtunder, and the Richmond obtunder. These are instruments and methods which use an elevation of temperature, and the vapor of an alcohol or any essential oil, or a combination of these, upon the dentine. The effect of throwing a blast of hot air upon a tooth is to heat it. By heating the tooth, evaporation of its dentine takes place. The fibril is drying up, and there is an outward movement as the water vaporizes and escapes from the tubule.

The application of a vaporizing agent in a warm or hot place is essentially one of desiccation. The heat is the only virtue in this, unless the agent has an affinity for water, when it may aid in carrying off the water vapor. The vapor of alcohol is effective on this account, and I think those who have recommended the use of oils or any agent which has not an affinity for water are laboring under a delusion.

Unfortunately, the agents which are most effective are most dangerous to the pulp, and in our selection of an agent for the case at hand this is to be borne in mind.

*The Chairman.*—Gentlemen, before adjournment, a little miscellaneous business has to be done. The chairman will announce the committee on necrology:—J. Taft, Louis Jack, George W. McElhaney, Frank Abbott, and Eugene S. Talbot.

Also the committee to fill the vacancies on the examination of the books of the Dental Protective Association, in place of Frank H. Garner and J. P. Wilson:—L. D. Shepard and H. A. Smith.

Adjourned.

